# Supplementary material for: Harmonizing heterogeneous single-cell gene expression data with individual-level covariate information
Source: Bioinform Adv. 2025 Aug 9;5(1):vbaf189. doi: 10.1093/bioadv/vbaf189 (PMC12380451; doi:10.1093/bioadv/vbaf189)
Supplement: vbaf189_Supplementary_Data [file vbaf189_supplementary_data.pdf]

Supplemenmtary File for Harmonizing Heterogeneous Single-cell Gene Expression Data with Individual-level Covariate Information

Yudi Mu[1] and Wei Vivian Li[1]

[1]Department of Statistics, University of California Riverside, Riverside, CA 92521, USA

Supplementary Methods

Optimization procedure in scINSIGHT2

Given the objective function $Q(\Theta')$ derived in Methods, the gradient and Hessian matrix of u_i are

$$\frac{\partial Q}{\partial u_i} = -\sum_{j=1}^{M} (y_{ij} - \mu_{ij}) \lambda_j + \frac{1}{\sigma^2} u_i,$$

$$\frac{\partial^2 Q}{\partial u_i \partial u_i^\top} = \sum_{j=1}^{M} \mu_{ij} \lambda_j \lambda_j^\top + \frac{1}{\sigma^2} I_P \quad \text{and} \quad \frac{\partial^2 Q}{\partial u_i \partial u_k^\top} = 0 \text{ for } k \neq i.$$

Similarly, for β_j and λ_j, the gradients and Hessian matrices are

$$\frac{\partial Q}{\partial \beta_j} = -\sum_{i=1}^{N} (y_{ij} - \mu_{ij}) x_i,$$

$$\frac{\partial Q}{\partial \lambda_j} = -\sum_{i=1}^{N} (y_{ij} - \mu_{ij}) u_i,$$

and

$$\frac{\partial^2 Q}{\partial \beta_j \partial \beta_j^\top} = \sum_{j=1}^{M} \mu_{ij} x_i x_i^\top,$$

$$\frac{\partial^2 Q}{\partial \lambda_j \partial \lambda_j^\top} = \sum_{j=1}^{M} \mu_{ij} u_i u_i^\top,$$

$$\frac{\partial^2 Q}{\partial \beta_j \partial \lambda_j^\top} = \sum_{j=1}^{M} \mu_{ij} x_i u_i^\top,$$

$$\frac{\partial^2 Q}{\partial \beta_j \partial \beta_k^\top} = \frac{\partial^2 Q}{\partial \lambda_j \partial \lambda_k^\top} = \frac{\partial^2 Q}{\partial \beta_j \partial \lambda_k^\top} = 0 \text{ for } k \neq j.$$

In addition, to update the standard deviation σ, we have

$$\frac{\partial Q}{\partial \sigma} = -\frac{\sum_{i=1}^N u_i^\top u_i}{\sigma^3} + \frac{NP}{\sigma},$$

$$\frac{\partial^2 Q}{\partial \sigma^2} = \frac{3\sum_{i=1}^N u_i^\top u_i}{\sigma^4} - \frac{NP}{\sigma^2}.$$

With the derived gradients and Hessian matrices, the estimation process can be summarized as the following steps:

(1) Initialize U, B, Λ randomly, where $B = (\beta_1, \beta_2, \ldots, \beta_m) \in \mathbb{R}^D$. Randomly sample $\beta_j \sim N(0, 0.1 \times I_M)$, $u_i \sim N(0, 0.1 \times I_P)$, $\lambda_j \sim N(0, 0.1 \times I_P)$ $(i = 1, ..., N, j = 1, ..., M)$. Initialize $\sigma = \frac{\sum_{i=1}^N \text{SD}(u_i)}{N}$.

(2) Repeat the following steps to perform updates until the stopping rule is satisfied or maximum iteration number (default to 5000) is reached.

$$U_{ik}^{(t+1)} = u_i^{(t)} - \alpha \left[\nabla_\theta^2 Q\left(u_i^{(t)}\right)\right]^{-1} \nabla_\theta Q\left(u_i^{(t)}\right),$$
$$\lambda_j^{(t+1)} = \lambda_j^{(t)} - \alpha \left[\nabla_\theta^2 Q\left(\lambda_j^{(t)}\right)\right]^{-1} \nabla_\theta Q\left(\lambda_j^{(t)}\right),$$
$$\beta_j^{(t+1)} = \beta_j^{(t)} - \alpha \left[\nabla_\theta^2 Q\left(\beta_j^{(t)}\right)\right]^{-1} \nabla_\theta Q\left(\beta_j^{(t)}\right),$$
$$\sigma^{(t+1)} = \sigma^{(t)} - \alpha(\frac{\partial^2 Q}{\partial(\sigma^{(t)})^2})^{-1} \frac{\partial Q}{\partial \sigma^{(t)}}.$$

(3) We compare the estimated cell embeddings at two adjacent steps using $\text{RMSE}_U(t) \triangleq \frac{1}{NP}\left(\sum_{i=1}^N \sum_{p=1}^P (U_{ip}^{(t+1)} - U_{ip}^{(t)})^2\right)^{\frac{1}{2}}$. The estimation is considered converged if $\text{RMSE}_U(t) < \epsilon_1$ and the average RMSE of the previous 100 iterations $\frac{1}{100}\sum_{q=t-99}^t \text{RMSE}_U(q) < \epsilon_2$, where ϵ_1 and ϵ_2 are sufficiently small values.

(4) Perform a rotation on the final estimates $\hat{U}$ and $\hat{\Lambda}$ for parameter identifiability. Denote the covariance matrix of $\hat{U}$ as S. We first obtain a matrix W such that $W^\top W = S^{-1}$, using the Cholesky algorithm. Then, we perform the following transformations: $\hat{U} \leftarrow \hat{U}W$ and $\hat{\Lambda} \leftarrow W^{-1}\hat{\Lambda}$. Then, we find a QR decomposition of $\hat{\Lambda}$ such that $\hat{\Lambda} = QR$, and update $\hat{U}$ and $\hat{\Lambda}$: $\hat{U} \leftarrow \hat{U}Q$ and $\hat{\Lambda} \leftarrow R$. This step ensures that $\hat{\Lambda}$ is a upper triangular matrix. Denote vector $d = \text{diag}(\hat{\Lambda})$, vector $s = \text{sign}(d)$, we get $\hat{U} \leftarrow (s\hat{U}^\top)^\top$, $\hat{\Lambda} \leftarrow s\hat{\Lambda}$. This will make $\hat{\Lambda}$ have positive diagonals.

Normalization of estimated cell embeddings

Since our estimation process relies on the Laplace approximation and may not directly yield cell embeddings that are optimal for clustering, we implement a normalization procedure from scINSIGHT to further refine the cell embeddings before clustering analysis.

(1) We use $\hat{U}_1 \in \mathbb{R}_{n_1 \times M}, \ldots, \hat{U}_K \in \mathbb{R}_{n_K \times M}$ to represent the estimated cell embeddings of the K samples. Each row of $\hat{U}_k$ $(k = 1, \ldots, K)$ is normalized so that its L_2 norm is 1. We denote the normalized matrices by $\tilde{U}_k$.

(2) For each pair of two samples, we find 20 nearest neighbours of each cell in one sample from the other sample based on $\tilde{U}_k$'s. Then, a mutual nearest neighbour graph of all cells is constructed based on the mutual nearest neighbours between every pair of samples.

(3) The Louvain method is used to perform clustering on the mutual nearest neighbour graph obtained in the previous step. Within each cluster, we perform the quantile normalization on $\tilde{U}_k$'s to reduce batch effects within clusters.

Challenges with the Negative Binomial distribution

Assume $y_{ij} \sim$ NB(μ_{ij}, θ_j), where θ_j is the inverse dispersion parameter for gene j. The prior over u_i remains N$(0, \sigma^2 I_p)$. The marginal log-likelihood function after integrating out the cell embeddings is

$$\ell(\Theta) = \sum_{i=1}^{N} \ell_i(\Theta) = \sum_{i=1}^{N} \log\left(\int \prod_{j=1}^{M} f\left(y_{ij} \mid u_i, \Theta\right) \pi\left(u_i\right) du_i\right)$$

The exponential of the log-likelihood of each cell is

$$\begin{aligned}
\exp\left(\ell_i(\Theta)\right) &= \int \prod_{j}^{M} \frac{\Gamma\left(y_{ij} + \theta_j\right)}{\Gamma\left(y_{ij} + 1\right)\Gamma\left(\theta_j\right)} \left(\frac{\mu_{ij}}{\mu_{ij} + \theta_j}\right)^{y_{ij}} \left(\frac{\theta_j}{\mu_{ij} + \theta_j}\right)^{\theta_j} \cdot \frac{1}{(2\pi)^{P/2} |\sigma^2 I|^{1/2}} \exp\left\{-\frac{u_i^\top u_i}{2\sigma^2}\right\} du_i \\
&\propto \int \exp\left\{\sum_{j=1}^{M} \left[\log\Gamma\left(y_{ij} + \theta_j\right) - \log\Gamma\left(y_{ij} + 1\right) - \log\Gamma\left(\theta_j\right)\right.\right. \\
&+ \left.\left. y_{ij} \log\left(\frac{\mu_{ij}}{\mu_{ij} + \theta_j}\right) + \theta_j \log\left(\frac{\theta_j}{\mu_{ij} + \theta_j}\right)\right] - \frac{1}{2\sigma^2} u_i^\top u_i - P\log(\sigma)\right\} du_i \\
&\triangleq \int \exp\{-G_i\left(u_i\right)\} du_i.
\end{aligned}$$

To approximate the intractable integral $\int \exp\left(-G_i\left(u_i\right)\right) du_i$, apply Laplace's method:

$$\ell_i(\Theta) \approx C - \frac{1}{2} \log\left|\frac{\partial^2 G_i}{\partial u_i \partial u_i^\top}\right| - G_i\left(\tilde{u}_i\right),$$

where $\frac{\partial^2 G_i}{\partial u_i \partial u_i^\top} = \sum_{j=1}^{M} \left[\left(-\frac{y_{ij}}{\mu_{ij}^2} + \frac{y_{ij} + \theta_j}{\left(\mu_{ij} + \theta_j\right)^2}\right) \mu_{ij}^2 \lambda_j \lambda_j^\top\right] + \frac{1}{\sigma^2} I$; $\tilde{u}_i$ is the solution to $\partial G_i(u)/\partial u_i = 0$ (i.e., $\tilde{u}_i = \arg\min G_i(u_i)$); C is a constant that does not depend on $\tilde{u}_i$. Then the log-likelihood

becomes

$$
\begin{aligned}
\ell_i(\Theta) \propto & -\frac{1}{2} \log \left| \sum_{j=1}^{M} \left[\left(-\frac{y_{ij}}{\mu_{ij}^2} + \frac{y_{ij} + \theta_j}{(\mu_{ij} + \theta_j)^2} \right) \mu_{ij}^2 \lambda_j \lambda_j^\top \right] + \frac{1}{\sigma^2} I \right| \\
& + \sum_{j=1}^{M} \left[\log \Gamma(y_{ij} + \theta_j) - \log \Gamma(y_{ij} + 1) - \log \Gamma(\theta_j) \right. \\
& \left. + \, y_{ij} \log \left(\frac{\mu_{ij}}{\mu_{ij} + \theta_j} \right) + \theta_j \log \left(\frac{\theta_j}{\mu_{ij} + \theta_j} \right) \right] - \frac{1}{2\sigma^2} u_i^\top u_i - P \log(\sigma).
\end{aligned}
$$

Then, we could define the objective function below to approximate the maximum likelihood estimation problem:

$$
Q(\Theta) = -\ell(\Theta) = -\sum_{i=1}^{N} \ell_i(\Theta).
$$

There are several challenges associated with solving the optimization problem under a negative binomial model. The objective function derived from the negative binomial likelihood is more complex than that of the Poisson model, and its non-convexity adds another layer of difficulty. Gradient computations reveal that the updates are highly sensitive to the gene-specific dispersion parameters, requiring optimization algorithms that are robust to such sensitivity. Moreover, the presence of gamma functions in multiple terms further increases computational cost. Due to these challenges, we opted to use the Poisson model in the current work.

Supplementary Figures

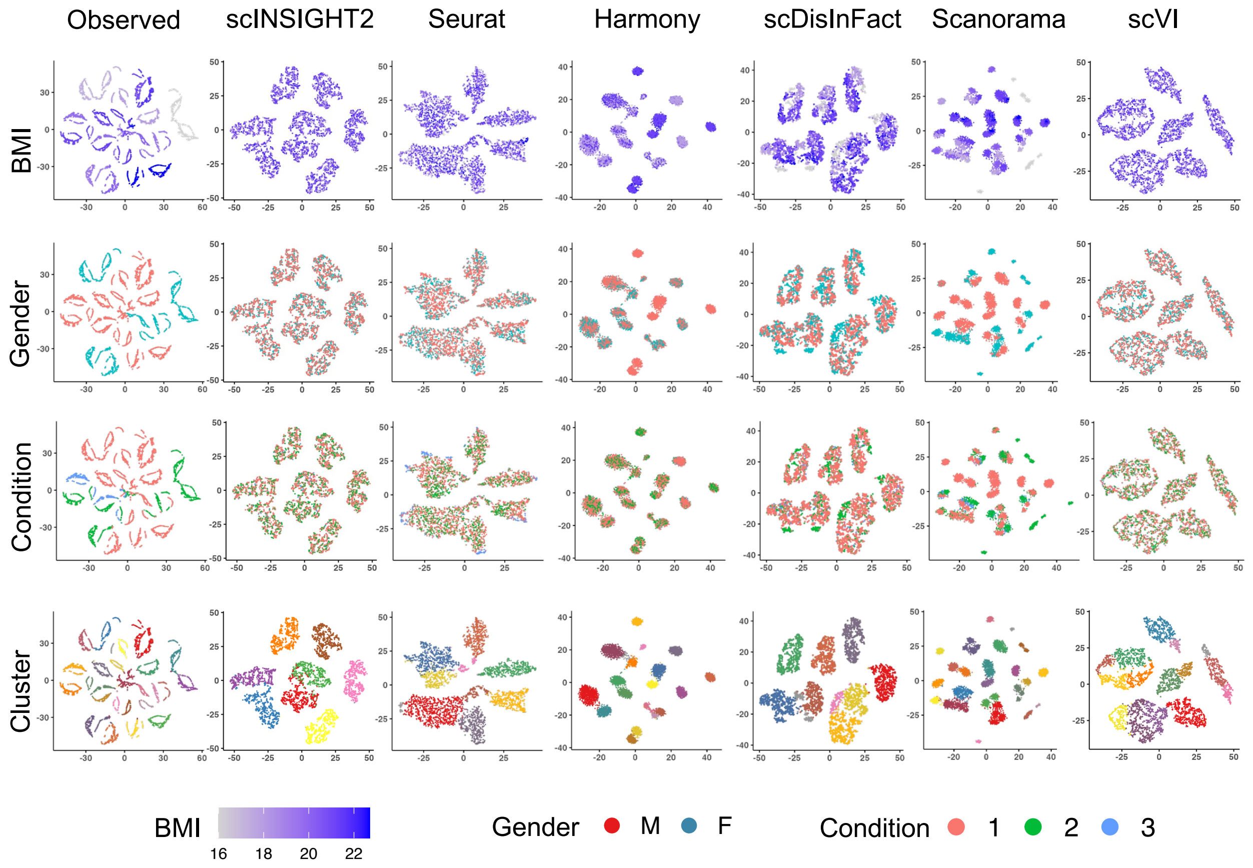

Figure S1: Comparison between integration methods in the simulation study (under first setting). For each method, tSNE plots with cells colored by BMI, gender, condition and inferred cluster are displayed.

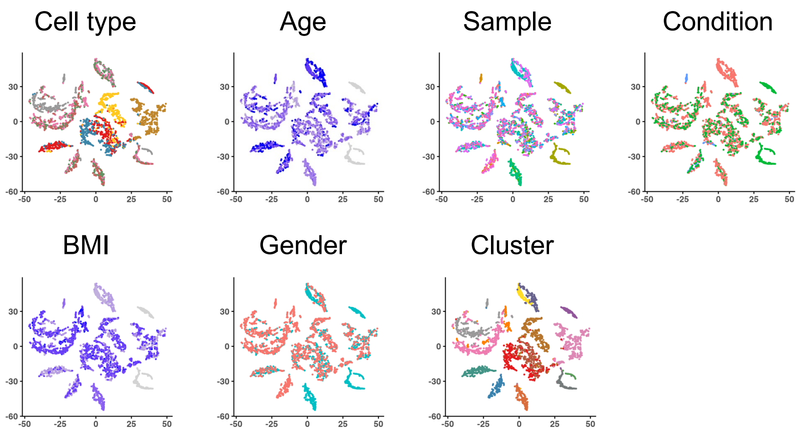

Figure S2: Integration result of scINSIGHT. The tSNE plots are colored by cell type, age, sample, condition, BMI, gender, and cluster.

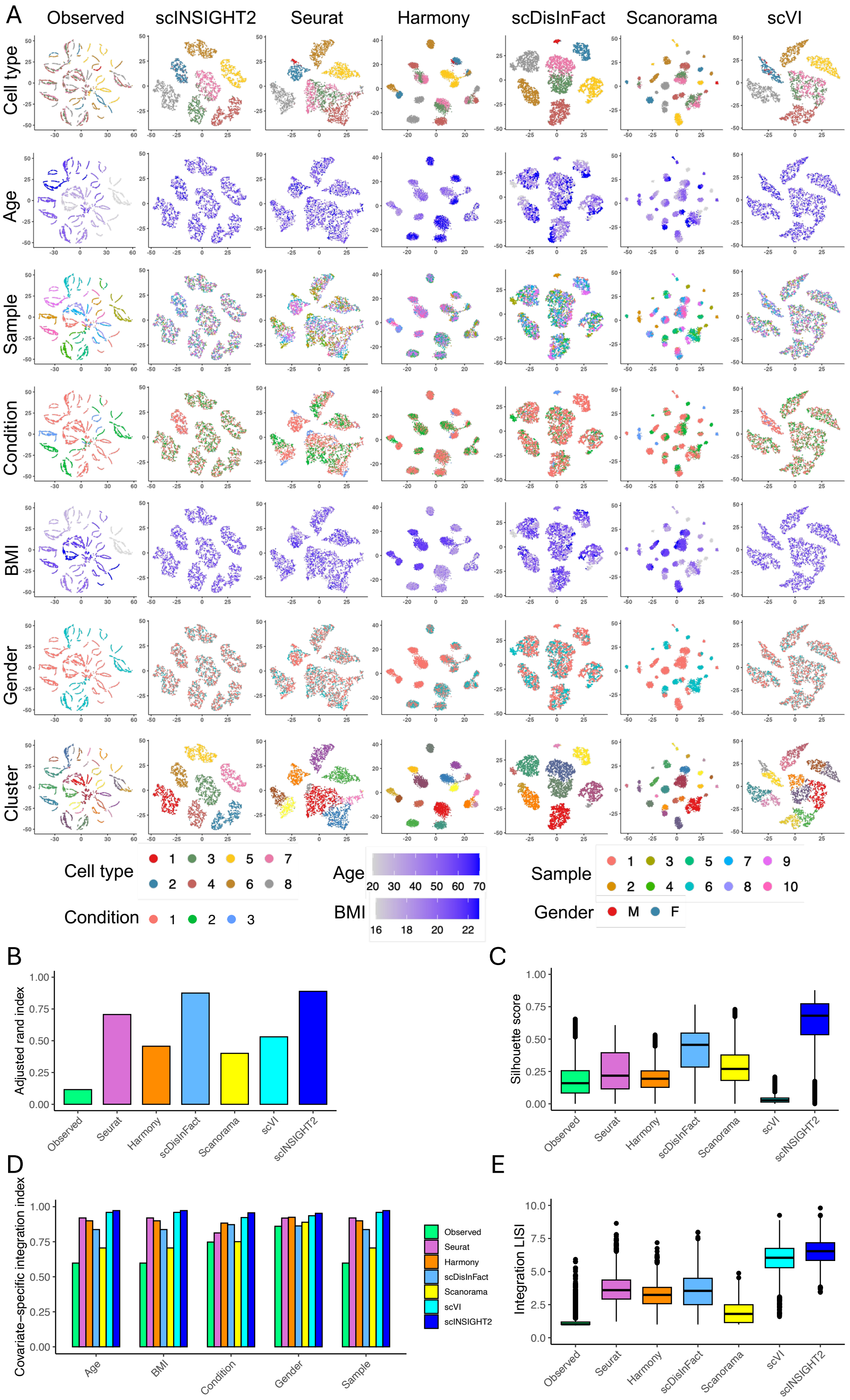

Figure S3: Comparison between integration methods in the simulation study with unique cell types. (**A**) tSNE plots of observed data and integrated data by different methods. For each method, tSNE plots colored by cell type, age, sample, condition, BMI, gender and cluster are displayed. (**B**) Adjusted Rand index calculated using clusters identified from the observed or integrated data. (**C**) Silhouette scores of the observed and integrated datasets. (**D**) Covariate-specific integration index of the observed and integrated data. (**E**) Integration LISI (iLISI) of the observed and integrated datasets.

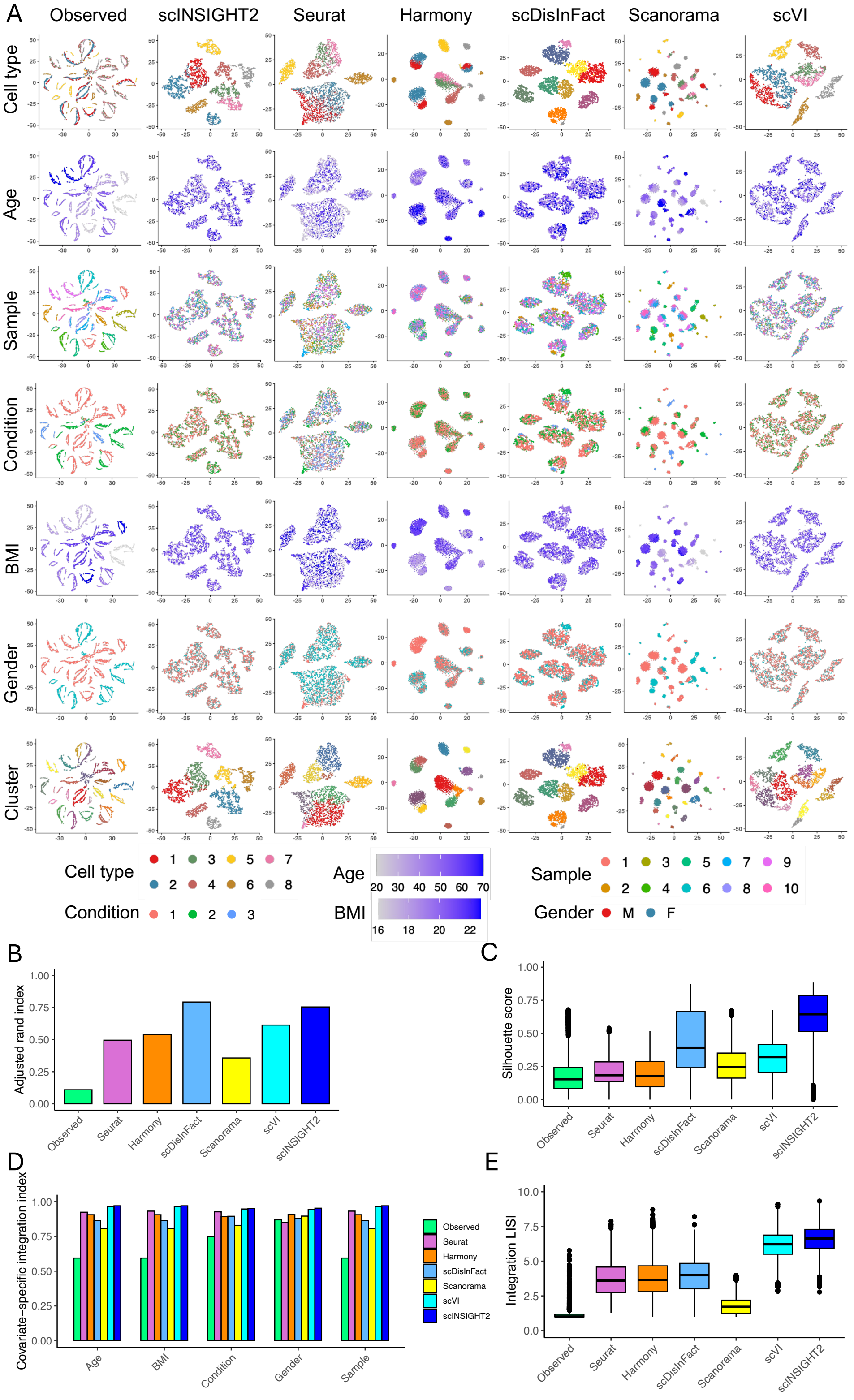

Figure S4: Comparison between observed data and different integration methods in the simulation study with age-associated cell types. (**A**) tSNE plots of observed data and integrated data by different methods. For each method, tSNE plots colored by cell type, age, sample, condition, BMI, gender and cluster are displayed. (**B**) Adjusted Rand index calculated using clusters identified from the observed or integrated data. (**C**) Silhouette scores of the observed and integrated datasets. (**D**) Covariate integration index of the observed and integrated data. (**E**) Integration LISI (iLISI) of the observed and integrated datasets.

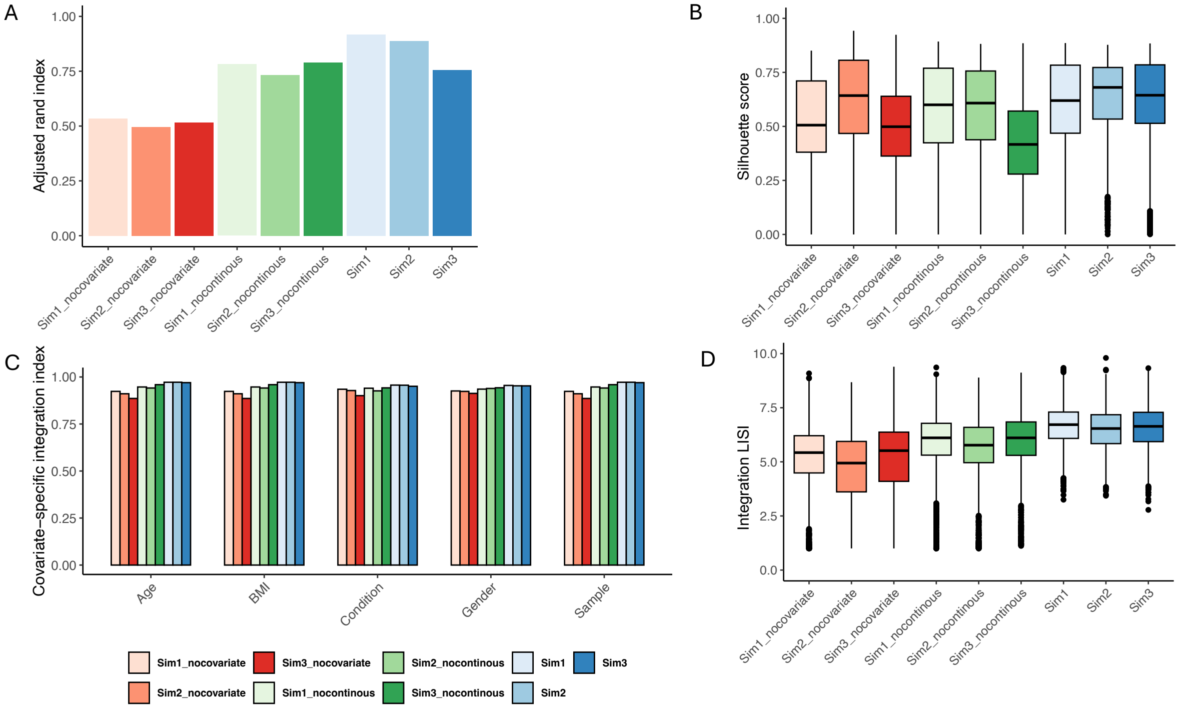

Figure S5: Comparison between full and reduced scINSIGHT2 models on simulated data. Sim1, Sim2, and Sim3 refer to the three simulation settings described in Methods. The abbreviations “nocovariate” and “nocontinuous” refer to the reduced scINSIGHT2 models excluding all covariates or excluding “continuous covariates, respectively. **(A)** Adjusted Rand index. **(B)** Silhouette scores. **(C)** Covariate-specific integration index. **(D)** Integration LISI (iLISI).

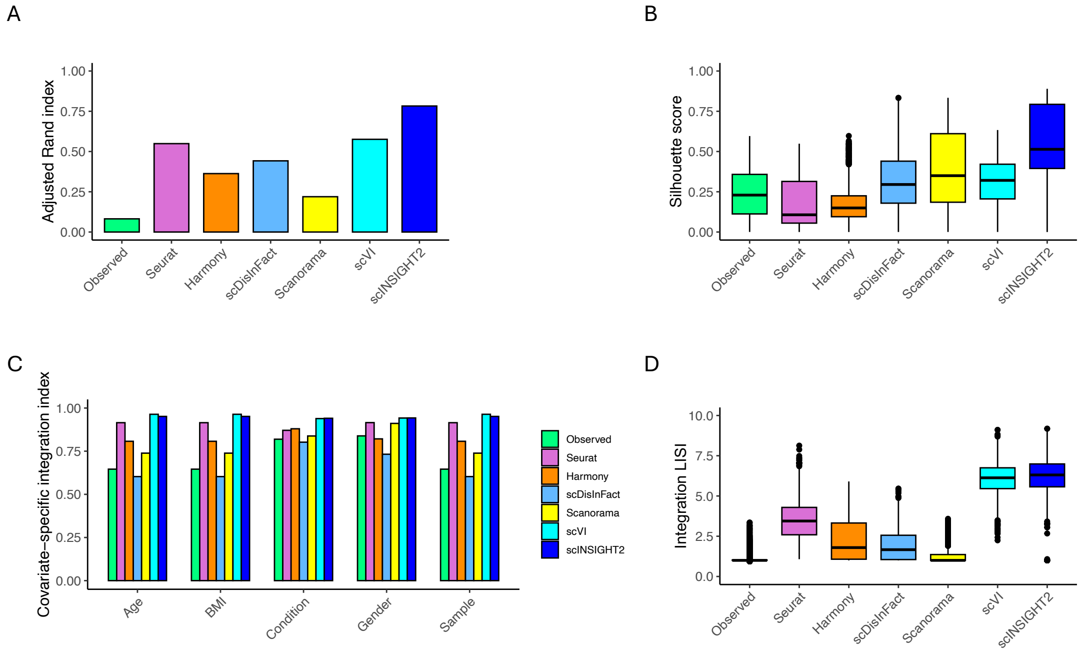

Figure S6: Comparison between integration methods in the simulation setting 1, with absolute values of covariates’ coefficients following U(1.5, 2). **(A)** Adjusted Rand index calculated using clusters identified from the observed or integrated data. **(B)** Silhouette scores of the observed and integrated datasets. **(C)** Covariate-specific integration index of the observed and integrated data. **(D)** iLISI of the observed and integrated datasets.

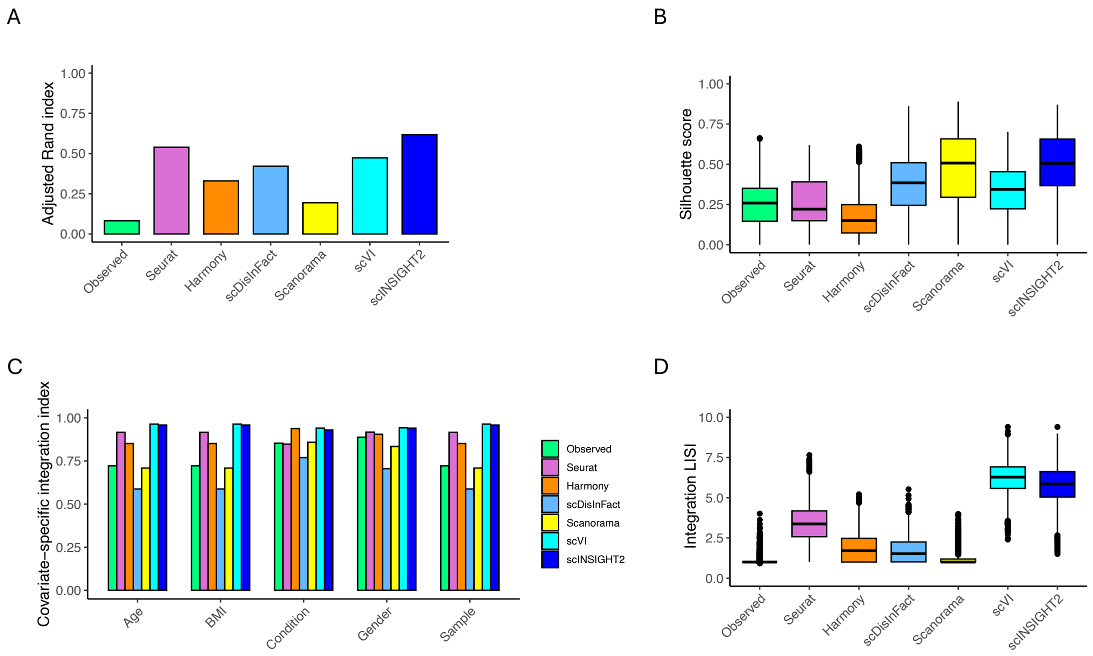

Figure S7: Comparison between integration methods in the simulation setting 1, with absolute values of covariates' coefficients following U(2, 2.5). **(A)** Adjusted Rand index calculated using clusters identified from the observed or integrated data. **(B)** Silhouette scores of the observed and integrated datasets. **(C)** Covariate-specific integration index of the observed and integrated data. **(D)** iLISI) of the observed and integrated datasets.

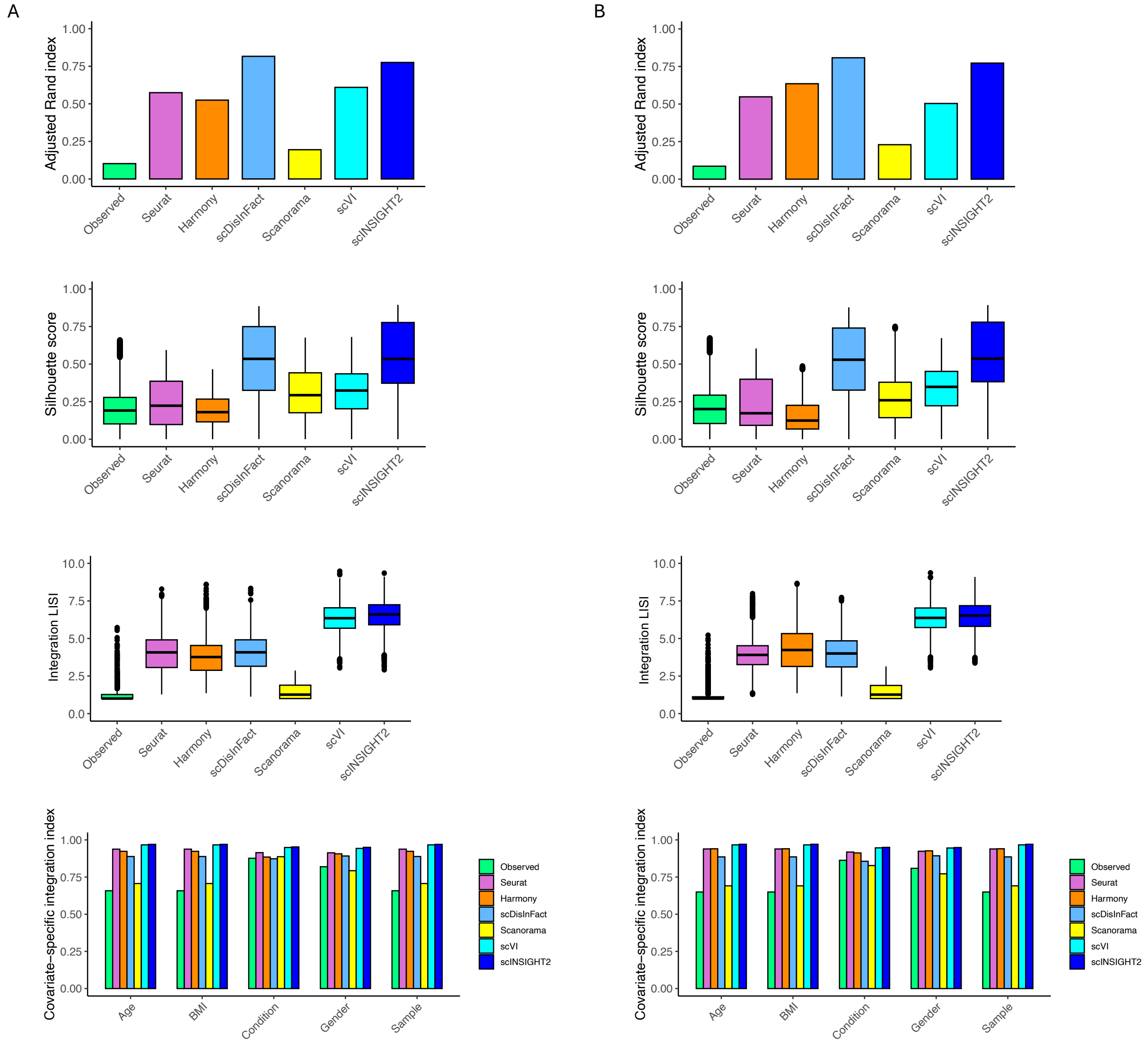

Figure S8: Comparison between integration methods in the simulation study where age and disease condition (severity) are uncorrelated **(A)** or correlated **(B)**. Each column shows ARI scores, silhouette scores, iLISI scores, and covariate-specific integration indices of the observed and integrated datasets from the top to the bottom.

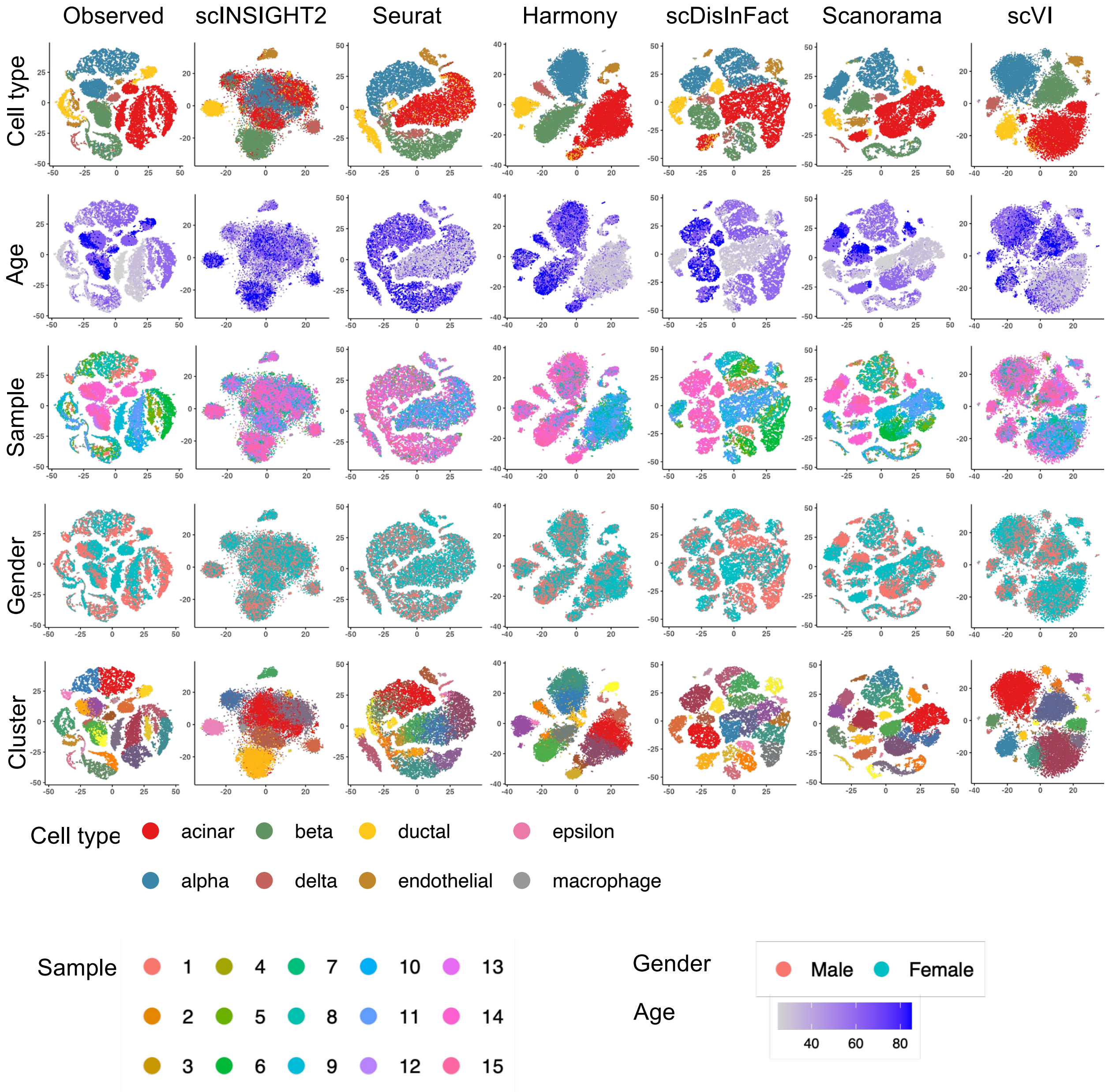

Figure S9: Comparison between observed data and integrated data by different integration methods in the pancreatic study. For each method, tSNE plots colored by cell type, age, sample, gender and cluster are displayed.

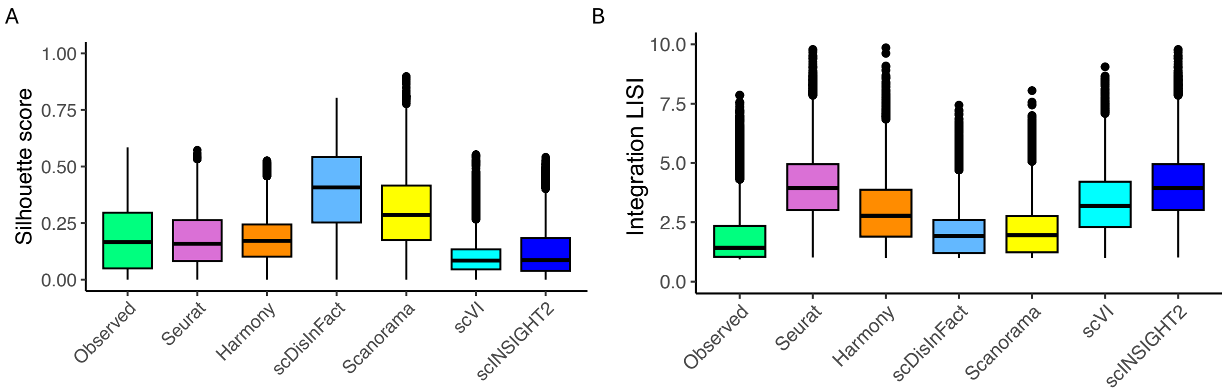

Figure S10: Comparison between observed data and integrated data by different integration methods in the pancreatic study. (**A**) Silhouette scores of the observed and integrated datasets. (**B**) Integration LISI (iLISI) of the observed and integrated datasets.

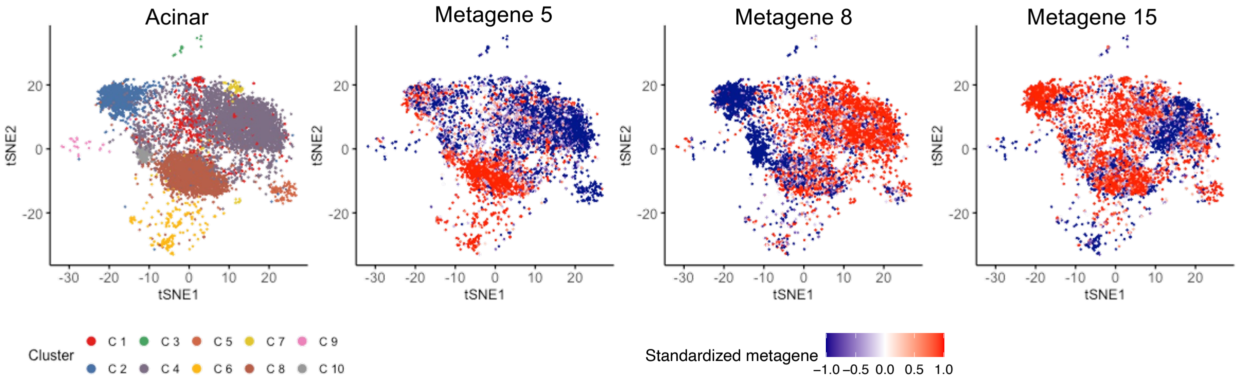

Figure S11: tSNE plots of acinar cells based on integrated data by scINSIGHT2. From left to right, the plots are colored by cluster and cell scores corresponding to metagene 5, metagene 8, and metagene 15.

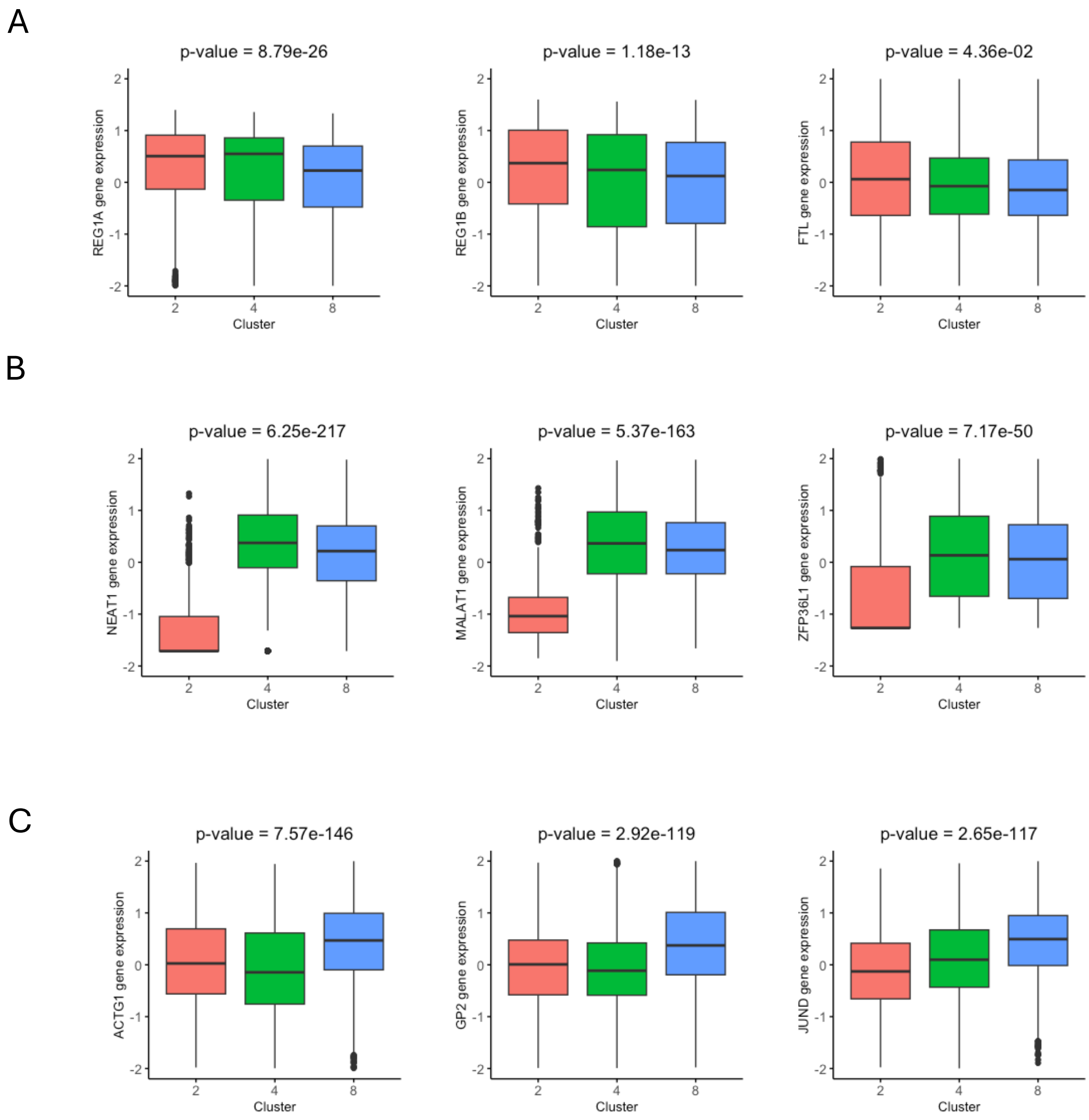

Figure S12: Top three marker genes of clusters C2, C4 and C8 in acinar cells. The P-values from Wilcoxon test are shown on the top of each figure. (**A**) Boxplots of top three marker genes upregulated in cluster C2. (**B**) Boxplots of top three marker genes upregulated in cluster C4. (**C**) Boxplots of top three marker genes upregulated in cluster C8.

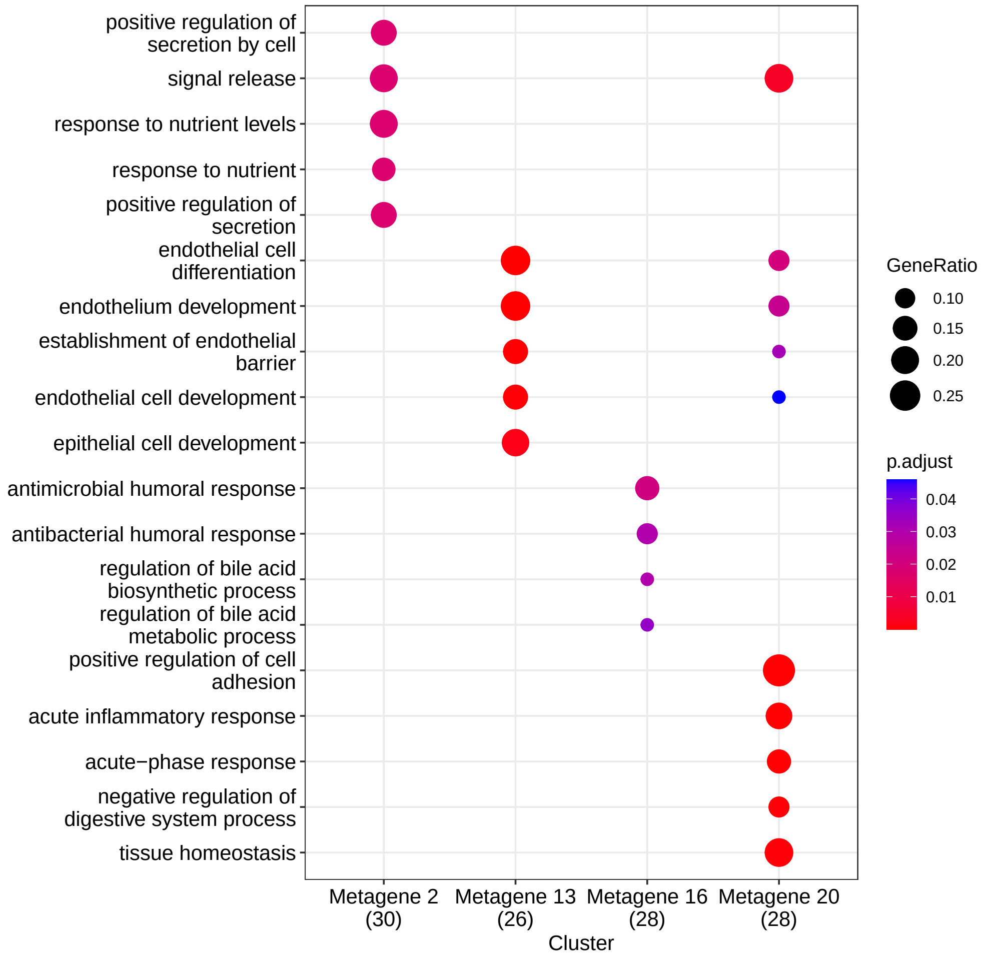

Figure S13: Enriched GO terms of metagene 2, metagene 13, metagene 16, and metagene 20 from the pancreatic dataset.

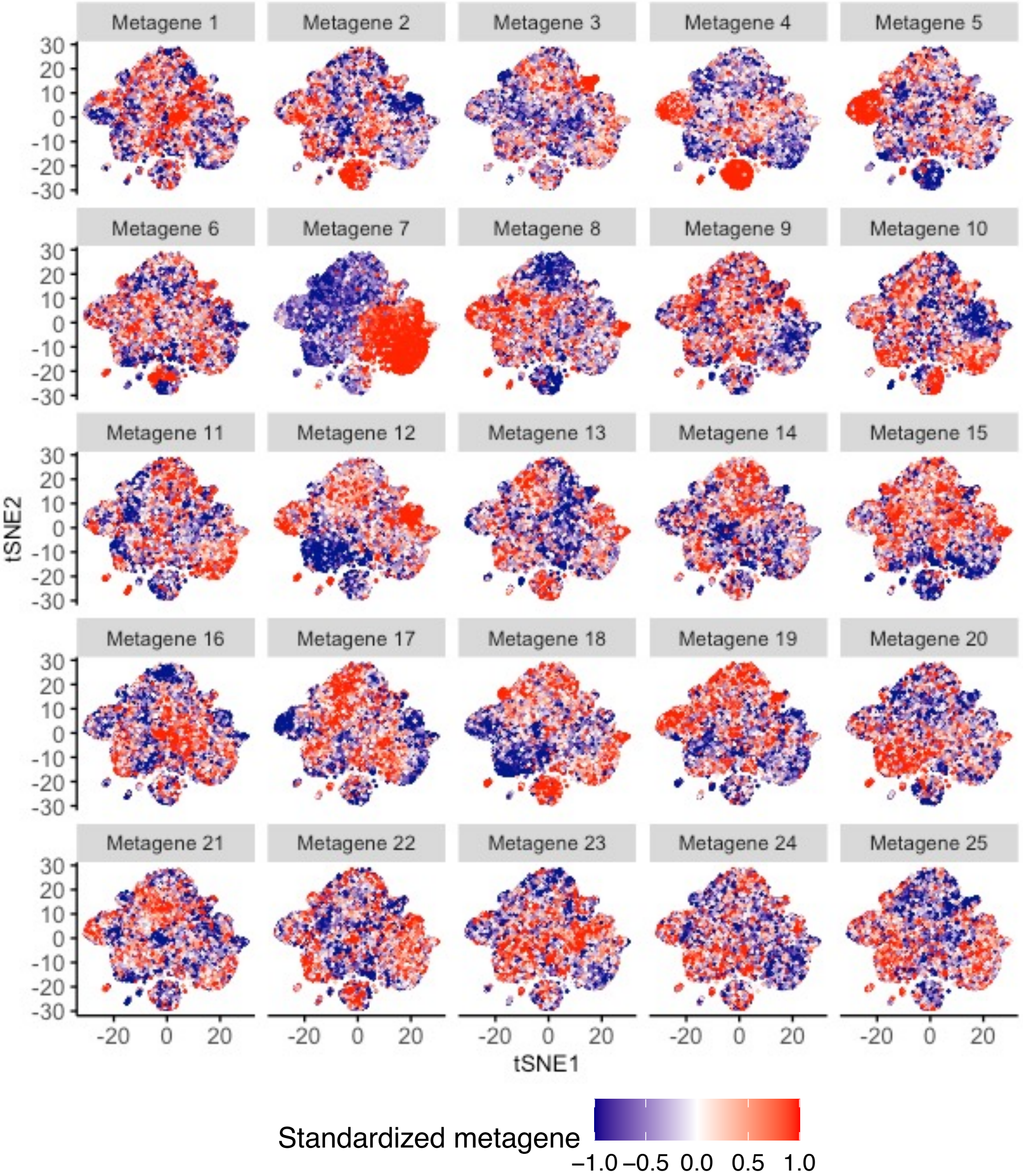

Figure S14: t-SNE plots based on integrated COVID-19 data processed by scINSIGHT2, with cells colored according to their normalized scores on metagenes.

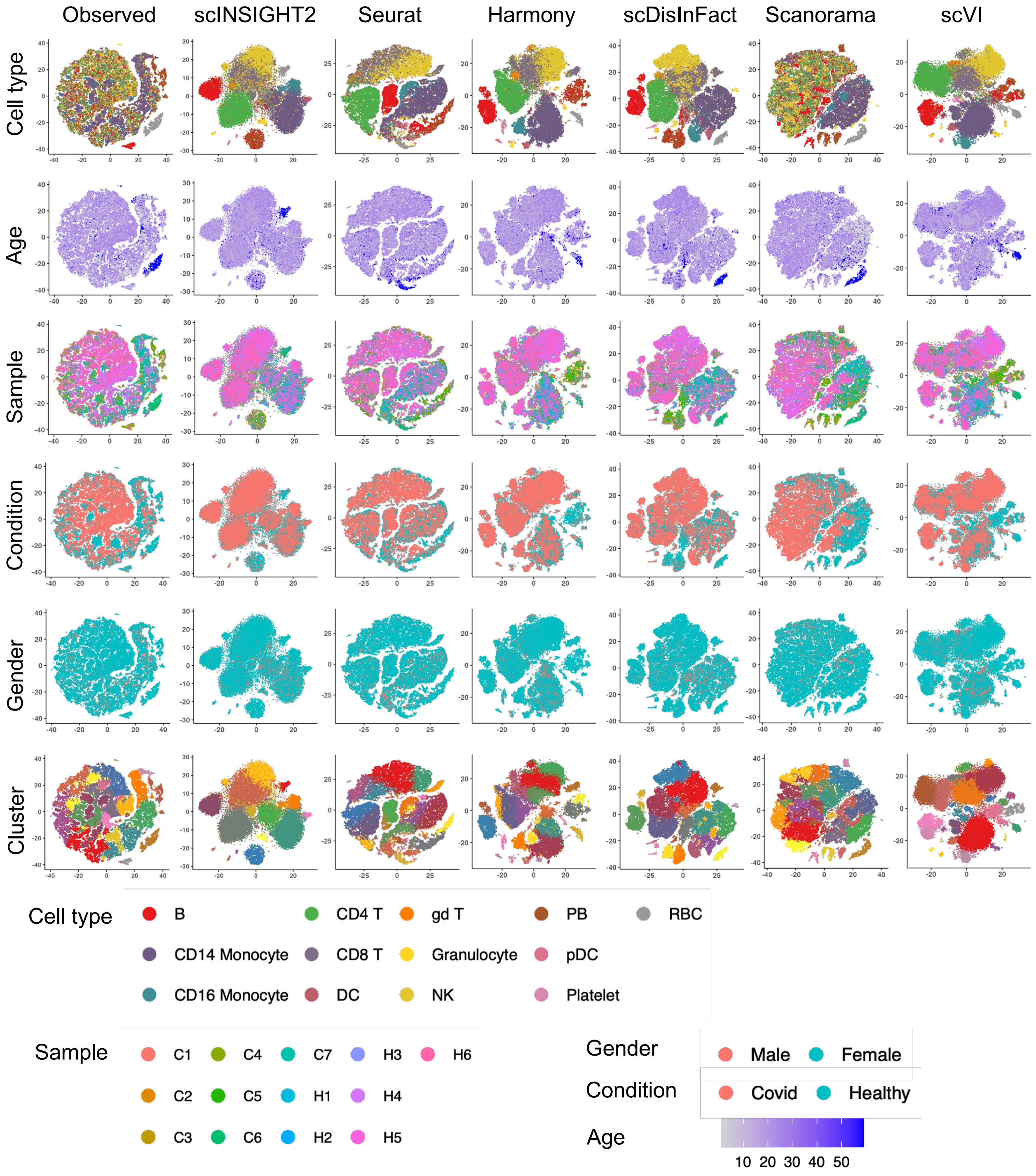

Figure S15: Comparison between observed data and different integration methods in the COVID-19 study. For each method, tSNE plots of observed data and integrated data colored by cell type, age, sample, condition, gender and cluster are displayed.

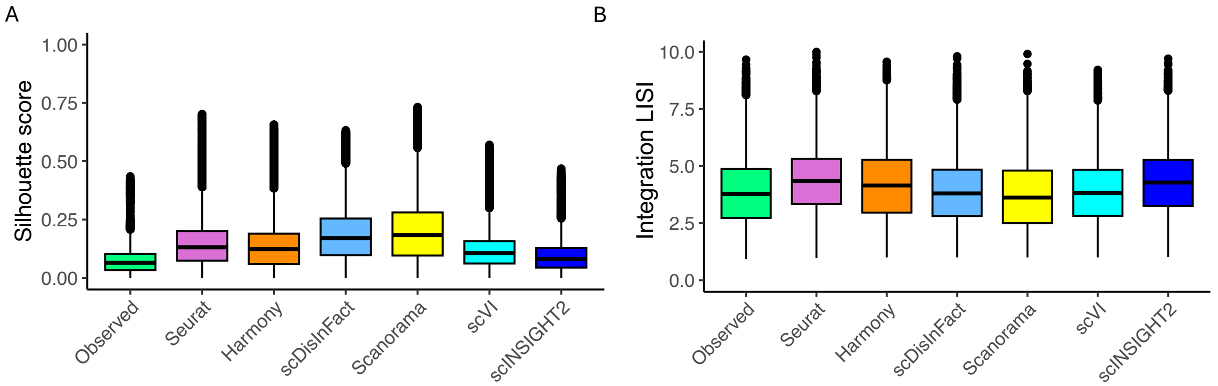

Figure S16: Comparison between observed data and integrated data by different integration methods in the COVID-19 study. (**A**) Silhouette scores of the observed and integrated datasets. (**B**) Integration LISI (iLISI) of the observed and integrated datasets.

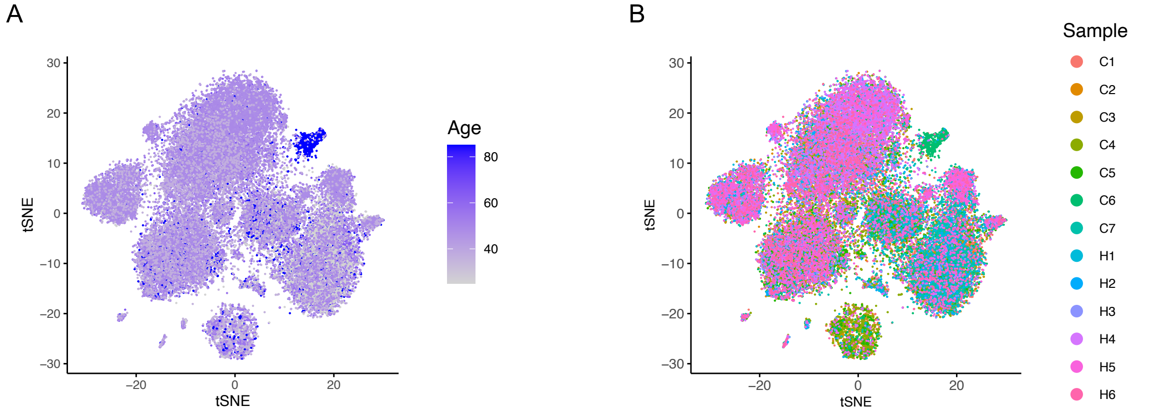

Figure S17: tSNE plots based on integrated COVID-19 data by scINSIGHT2, colored by age (**A**) or sample (**B**).

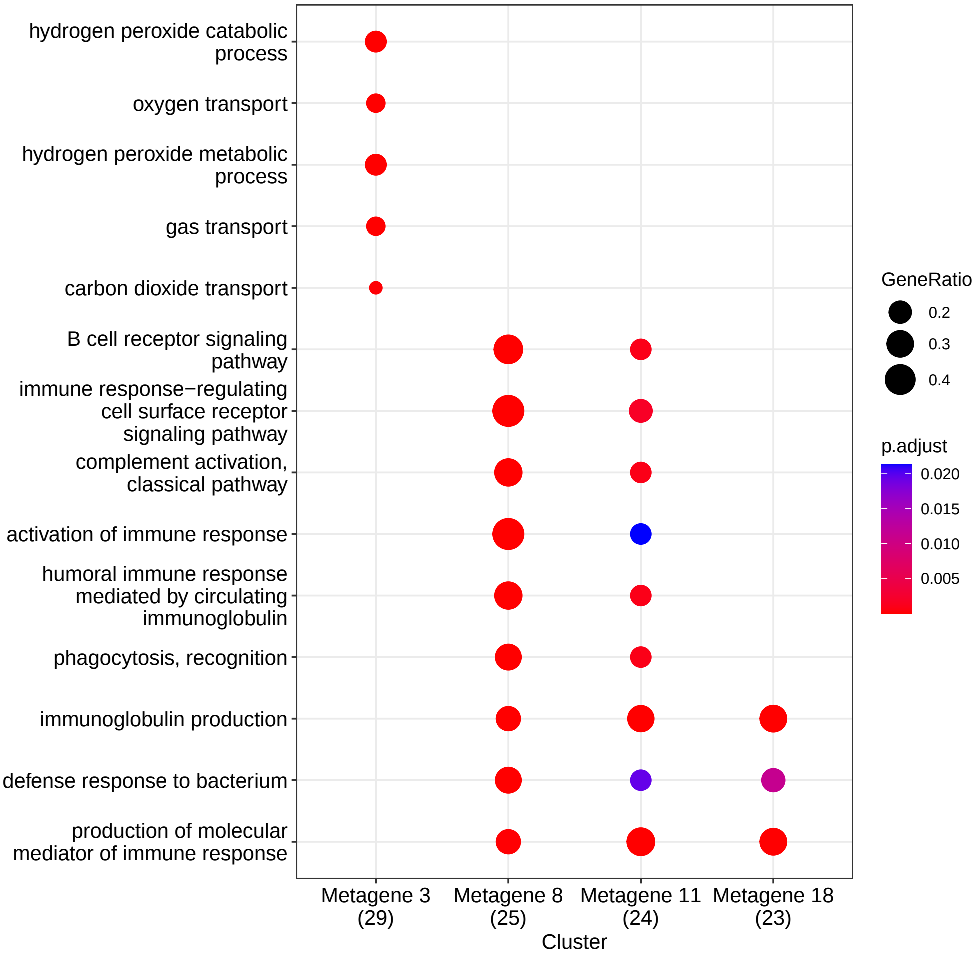

Figure S18: Enriched GO terms of metagene 3, metagene 8, metagene 11 and metagene 18 from the COVID-19 dataset.

Supplementary Tables

Table S1: Mean parameters of normal distribution for the simulation of cell embeddings.

cell type	p=1	p=2	p=3	p=4	p=5
type1	-1.2	-0.5	0	0.5	1.2
type2	-0.5	0	0	0.5	1.2
type3	1.2	0.5	0	-0.5	-1.2
type4	1.2	0.5	0	-1.2	-0.5
type5	-1.2	0	0.5	1.2	-0.5
type6	-1.2	0	0.5	-1.2	0.5
type7	1.2	0.5	0	0	-0.5
type8	0.5	0.5	0	-0.5	1.2

Table S2: Computational time and memory usage of scINSIGHT2 and alternative methods. The time and memory usage reported for scINSIGHT2 correspond to the optimal number of metagenes and initialization seed.

Dataset	scINSIGHT2	Seurat	Harmony	Scanorama	scVI	scDisInFact
Simulation<br>$5,000$ cells<br>10 samples<br>5 covariates	84 mins<br>2.01 GB	3.8 mins<br>1.4 GB	0.43 mins<br>0.919 GB	0.82 mins<br>0.367 GB	2.7 mins<br>2.2 GB	5.3 mins<br>0.746 GB
Pancreas<br>$27,435$ cells<br>15 samples<br>3 covariates	660 mins<br>9.56 GB	30 mins<br>3.26 GB	2.08 mins<br>1.537 GB	3.8 mins<br>3.32 GB	10.55 mins<br>3.63 GB	7.93 mins<br>0.88 GB
COVID-19<br>$44,721$ cells<br>13 samples<br>4 covariates	1680 mins<br>15.26 GB	71 mins<br>5.47 GB	3.54 mins<br>2.64 GB	36 mins<br>8.98 GB	11.6 mins<br>5.98 GB	12 mins<br>0.835 GB
